# ^18^F-florbetaben positron emission tomography detects cardiac involvement in systemic AA amyloidosis

**DOI:** 10.1007/s00259-020-04861-4

**Published:** 2020-05-20

**Authors:** Maria Papathanasiou, Alexander Carpinteiro, Tim Hagenacker, Ken Herrmann, Tienush Rassaf, Christoph Rischpler, Peter Luedike

**Affiliations:** 1 Department of Cardiology and Vascular Medicine, West German Heart and Vascular Center, University Hospital Essen, Hufelandstrasse 55, 45147 Essen, Germany; 2 West German Amyloidosis Center, University Hospital Essen, Hufelandstrasse 55, 45147 Essen, Germany; 3 Department of Hematology, University Hospital Essen, Hufelandstrasse 55, 45147 Essen, Germany; 4 Department of Molecular Biology, University of Duisburg-Essen, Hufelandstrasse 55, 45147 Essen, Germany; 5 Department of Neurology, University Hospital Essen, Hufelandstrasse 55, 45147 Essen, Germany; 6 Department of Nuclear Medicine, University Hospital Essen, Hufelandstrasse 55, 45147 Essen, Germany

A 62-year-old female with known systemic serum amyloid A (AA) amyloidosis presented with signs and symptoms of new-onset heart failure. Echocardiography demonstrated mild left ventricular (LV) dilation, preserved ejection fraction, and grade II diastolic dysfunction without typical signs of cardiac amyloidosis (CA) but a profound focal hypertrophy of the free right ventricular (RV) wall (a, b). Positron emission tomography/computed tomography (PET/CT) with the amyloid-binding tracer ^18^F-florbetaben was subsequently performed since endomyocardial biopsy was not deemed justified due to localized hypertrophy of the free RV wall. As shown in c-e, a highly increased RV tracer uptake with only moderately increased LV tracer uptake was found (retention index for RV and LV is 0.0026 and 0.0016, respectively). Tracer uptake co-localized with the echocardiographic finding of RV hypertrophy, suggesting cardiac involvement of AA amyloidosis with predominant right-sided amyloid deposition.

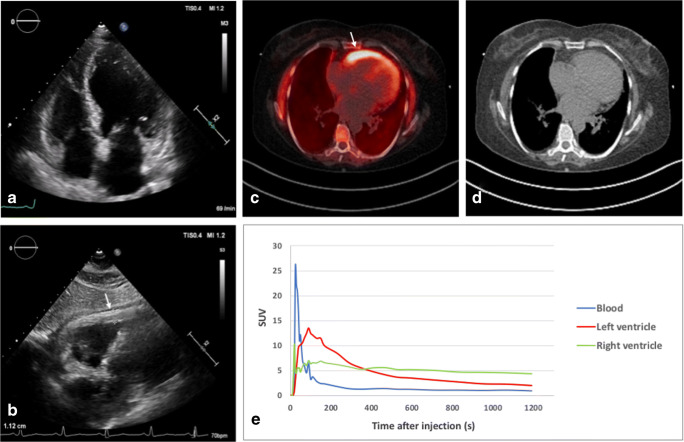



Amyloid-binding radiotracers have already received approval for beta-amyloid brain imaging. Previous exploratory studies demonstrated their high diagnostic accuracy for CA of transthyretin or light-chain type [1–3], yet no study has evaluated their utility in an AA amyloidosis cohort. While endomyocardial biopsy is an established method for diagnosing CA, it may be prone to sampling error in the case of localized disease. This report demonstrates the sensitivity of ^18^F-florbetaben PET/CT to detect AA-CA. PET/CT provides high image quality and quantitative measures of tracer uptake, thus making detection, localization, and absolute quantification of amyloid feasible and has the potential to substitute or even outperform endomyocardial biopsy, particularly in the case of focal or early-stage disease.

## Data Availability

Clinical and image data are available for review upon request.
